# Evaluation of +49 A>G (rs231775) Variant in CTLA4 Gene and SCTLA-4 Serum Levels in Plaque Psoriasis in a Mestizo Mexican Population

**DOI:** 10.3390/ijms27073202

**Published:** 2026-04-01

**Authors:** María Guadalupe Cortés-Ruiz, Katia Alejandra Wheber-Hidalgo, Brenda Fernanda Hernández-Nicols, Fernando Gabriel Buenrostro-Camacho, Jorge Hernández-Bello, Omar Graciano-Machuca, Anabell Alvarado-Navarro

**Affiliations:** 1 Centro de Investigación en Inmunología y Dermatología, Departamento de Fisiología, Centro Universitario de Ciencias de la Salud, Universidad de Guadalajara, Sierra Mojada 950, Colonia Independencia, Guadalajara 44340, Jalisco, Mexico; lup23062001@gmail.com (M.G.C.-R.); kwheberh@gmail.com (K.A.W.-H.); brendahernandeznicols@gmail.com (B.F.H.-N.); fer2015buenrostro@gmail.com (F.G.B.-C.); 2 Instituto de Investigación en Ciencias Biomédicas, Centro Universitario de Ciencias de la Salud, Universidad de Guadalajara, Sierra Mojada 950, Colonia Independencia, Guadalajara 44340, Jalisco, Mexico; jorge.hernandezbello@cucs.udg.mx; 3 Centro de Investigación en Procesamiento Digital de Señales, Centro Universitario de los Valles, Universidad de Guadalajara, Carretera a Guadalajara, Supermanzana El Km 45.5, Caimanero 46708, Jalisco, Mexico; omargmachuca@academicos.udg.mx

**Keywords:** plaque psoriasis, *CTLA4* variant, rs231775, soluble CTLA-4, immune checkpoint, genetic susceptibility

## Abstract

Plaque psoriasis (PP) is a chronic immune-mediated skin disorder characterized by T-cell dysregulation and an imbalance between regulatory T cells (Treg) and T helper 17 (Th17) cells. Cytotoxic T-lymphocyte-associated antigen 4 (CTLA-4), a key inhibitory checkpoint molecule expressed on Treg cells, and its soluble isoform (sCTLA-4) are critical regulators of peripheral immune tolerance and may contribute to PP pathogenesis. This case–control study evaluated the association between the +49 A>G variant of the *CTLA4* gene (rs231775) and susceptibility to PP in a mestizo population from western Mexico and assessed serum sCTLA-4 levels. A total of 204 patients with PP and 214 control subjects (CS) were genotyped using PCR-RFLP, and sCTLA-4 concentrations were measured by ELISA. The AG genotype was the most frequent in both groups (49% in PP and 53% in CS), with no significant differences in genotype or allele distributions. Serum sCTLA-4 levels were significantly higher in CS compared to patients (*p* < 0.05), and no genotype-dependent differences were observed. The rs231775 variant was not associated with PP susceptibility in this population. However, reduced circulating sCTLA-4 levels in patients suggest impaired CTLA-4-mediated immune regulation independent of this variant.

## 1. Introduction

Psoriasis is a chronic, non-contagious, inflammatory skin disease recognized worldwide by the World Health Organization [1] as a serious condition. Psoriasis affects approximately 100 million individuals worldwide [2] and around 2% of the Mexican population [3]. In the United States, the prevalence is 3.2% in adults and 0.13% in children [4]. Psoriasis is increasingly viewed as a systemic disease due to its association with comorbidities such as Crohn’s disease, metabolic syndrome, cardiovascular disease, depression, and cancer [5].

The pathogenesis of psoriasis is complex and involves multiple immunological, genetic, and environmental factors [6] which initiate the inflammatory signaling cascade, leading to alterations in keratinocyte differentiation and a chronic inflammatory process. This is characterized by Th1 cell activation and a Th17/Treg imbalance, with CTLA-4, an inhibitory receptor expressed on activated T cells. CTLA-4 binds to B7 (CD80/CD86) on antigen-presenting cells, preventing T lymphocyte activation and promoting peripheral tolerance [7]. Genetic defects or functional alterations in CTLA-4 may favor autoimmunity by enabling persistent T cell activation [8].

In psoriasis, the IL-23/IL-17 axis plays a central pathogenic role, driving keratinocyte hyperproliferation and sustained cutaneous inflammation [9]. Regulatory T cells (Treg) normally restrain excessive immune activation; however, impaired Treg suppressive function or reduced inhibitory signaling may facilitate Th17 expansion and chronic inflammation [10]. CTLA-4 competes with CD28 for binding to CD80/CD86, attenuating costimulatory signaling and downstream activation of pathways such as NF-κB. Reduced CTLA-4 expression or signaling efficiency may therefore enhance CD28-mediated T-cell activation and amplify Th17-driven responses, contributing to immune dysregulation in psoriasis [11].

The *CTLA4* gene, located on chromosome 2q33 [5], encodes two main transcripts: the full-length isoform with a transmembrane domain and the soluble isoform, which lacks this domain and is found as a soluble monomeric protein [7]. The *CTLA4* gene contains several variants, including 16 SNVs and a microsatellite, which determine its function. The +49 A>G (rs231775) variant has been most widely studied; it encodes a threonine-to-alanine substitution in the leader peptide, potentially altering protein trafficking and expression [12].

The soluble isoform of CTLA-4 (sCTLA-4) retains the ability to bind CD80/CD86 and may exert systemic immunomodulatory effects. Altered circulating levels of sCTLA-4 have been described in several autoimmune diseases, including rheumatoid arthritis [10] and systemic lupus erythematosus [13], suggesting that quantitative changes in this molecule may reflect impaired immune regulation. However, data regarding sCTLA-4 levels in psoriasis remain limited and inconsistent.

Although the +49 A>G polymorphism has been associated with susceptibility to multiple autoimmune disorders, its relationship with psoriasis has yielded conflicting results across different ethnic populations, and studies in Latin American or Mexican mestizo populations are scarce [14]. Moreover, the functional impact of this variant on circulating sCTLA-4 levels in psoriasis remains unclear.

Given CTLA-4’s immunoregulatory role, this study aimed to evaluate the association between the +49 A>G variant and PP susceptibility in a mestizo population from western Mexico.

## 2. Results

### 2.1. Clinical and Demographic Characteristics of the Study Groups

The clinical and demographic characteristics of the study groups are shown in Table 1. A total of 214 control subjects (CS) and 204 patients with plaque psoriasis (PP) were included. The mean age was 44.7 ± 12.06 years in the CS group and 46.8 ± 14.48 years in the PP group, with no significant difference between groups (*p* = 0.078).

Sex distribution was comparable, with males slightly predominating in both groups (CS: 51%; PP: 53%; *p* = 0.77).

The mean disease duration among patients with plaque psoriasis was 9 ± 10 years, and the mean age at disease onset was 38 ± 18 years.

### 2.2. Genotype and Allele Frequencies

The genotype frequency distribution of the variant was in Hardy–Weinberg equilibrium in the CS group (*p* > 0.05). We observed that the AG genotype was the most frequent in both study groups (PP 49% and CS 53%), without statistically significant differences (*p* = 0.30). Furthermore, genotypes with the dominant G allele are more common in CS (69%) than in PP (65%); however, these differences were not statistically significant (*p* = 0.33). Also, when analyzing the genetic model of recessive inheritance, we found no statistically significant difference (Table 2).

### 2.3. sCTLA-4 Serum Levels in Patients and Controls

**Figure 1:**
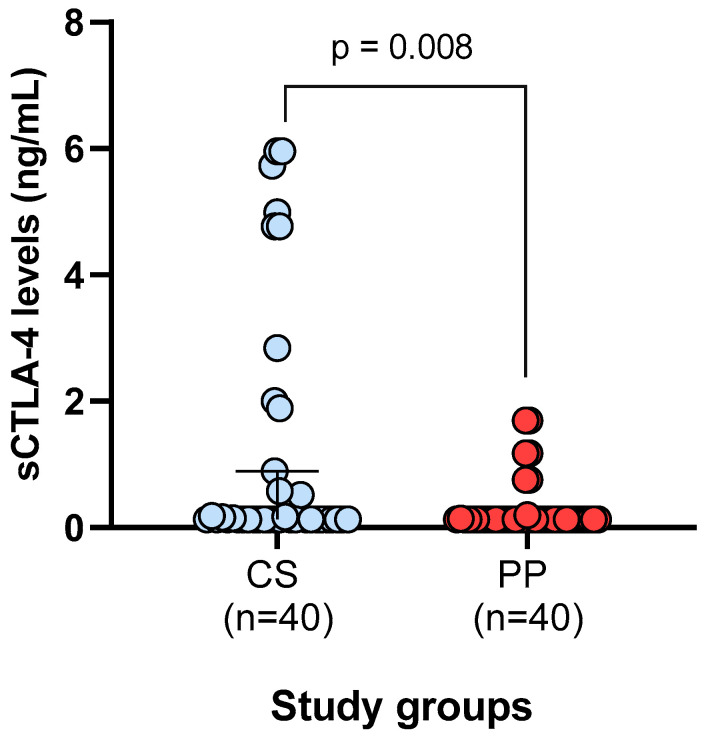
Comparison of sCTLA-4 serum levels between study groups. *p*-values were calculated using the Mann–Whitney U test. Data are presented as medians and interquartile ranges. Abbreviations: PP, plaque psoriasis; CS, control subjects. A substantial proportion of samples showed values at the assay’s lower detection limit (0.13 ng/mL).

When comparing sCTLA-4 serum levels between PP and CS, higher levels were observed in CS (1.27 ng/mL) than in PP (0.32 ng/mL), with a statistically significant difference (*p* = 0.008) (Figure 1).

Spearman correlation analysis was performed to evaluate the relationships among clinical, anthropometric, and immunological variables in patients with PP. A statistically significant positive correlation was observed between disease duration and PASI score (ρ = 0.51, *p* = 0.036). However, sCTLA-4 levels were not significantly correlated with PASI (ρ = 0.09, *p* = 0.73) or with any other clinical or anthropometric variables, including age, BMI, or disease duration (all *p* > 0.05) (Table 3).

### 2.4. +49 A>G Genotypes and sCTLA-4 Serum Levels

**Figure 2:**
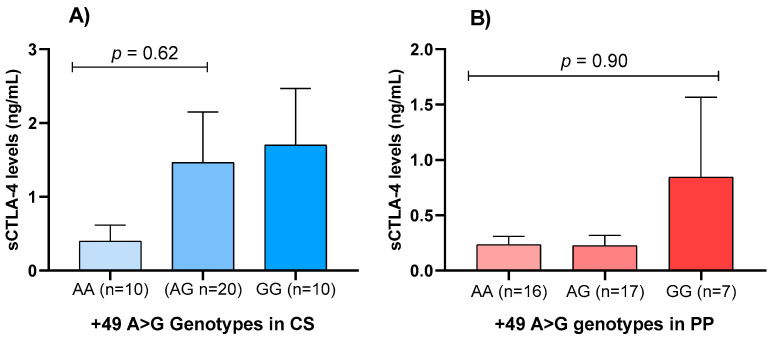
Distribution of sCTLA-4 levels across *CTLA4* gene genotypes. (**A**) sCTLA-4 levels by rs231775 genotype in controls. (**B**) sCTLA-4 levels by rs231775 genotype in PP. Statistical analysis was conducted using the Kruskal–Wallis test, followed by Dunn’s multiple comparisons test to assess differences across groups. The box plots display the distribution of data from the fifth to the ninety-fifth percentile. The central box represents the median and quartiles, while the whiskers extend to show the data’s range within these percentiles. Abbreviations: PP, plaque psoriasis; CS, control subjects.

Figure 2 shows the comparison of serum sCTLA-4 concentrations by genotype in both study groups. Among controls, individuals with the GG genotype had the highest mean sCTLA-4 levels (1.70 ± 2.16 ng/mL), followed by AG (1.46 ± 2.98 ng/mL) and AA (0.39 ± 0.61 ng/mL). A similar trend was observed among cases, with mean sCTLA-4 concentrations of 0.84 ± 1.77 for GG, 0.22 ± 0.37 for AG, and 0.23 ± 0.29 ng/mL for AA. However, the differences were not statistically significant (*p* > 0.05).

## 3. Discussion

The SNV +49 A>G of the *CTLA4* gene (rs231775) results in a threonine-to-alanine substitution within the leader peptide, potentially altering intracellular trafficking and reducing surface expression of CTLA-4 on T lymphocytes. Impaired expression of this inhibitory receptor may diminish negative regulatory signaling, favoring sustained T-cell activation and the emergence of autoreactive responses [15,16]. Functionally, reduced surface CTLA-4 may enhance CD28-mediated costimulatory signaling, promoting downstream activation of NF-κB and facilitating Th1/Th17 differentiation, pathways that are central to psoriasis immunopathogenesis [17].

This genetic variant has been widely studied in various autoimmune diseases, particularly in Caucasian and Asian populations but results have been inconsistent, such as rheumatoid arthritis [14,18], ankylosing spondylitis [19], systemic lupus erythematosus [20], type 1 diabetes [21], Hashimoto’s thyroiditis [22], Graves’ disease [23], and psoriasis [5].

In the present study, although the AG genotype was the most frequent among patients with plaque psoriasis, no significant association between the +49 A>G variant and psoriasis susceptibility was identified. These results align with findings from a meta-analysis [24]. Conversely, case–control studies conducted in Asian and Turkish populations have found an association between the G allele and psoriasis risk [5,25]. Psoriasis is a multifactorial disease that depends mainly on genetic predisposition and external environmental factors.

As a polygenic pathology, psoriasis has multiple alleles and risk loci that regulate the immune response [4]. It is plausible that the effect size of rs231775 is modest and may only become detectable in specific genetic backgrounds or in the presence of interacting loci within the HLA region or IL-23/IL-17 pathway genes. Differences across populations may arise from ethnic genetic structure. The Mexican Mestizo population is highly admixed, consisting of approximately 60–64% European, 21–25% Amerindian, and 15% African ancestry, with minor contributions from Asian ancestry [26,27,28]. This heterogeneity may modify allele frequencies, linkage patterns, and gene–environment interactions, potentially explaining some of the inconsistencies between our findings and those reported in other populations. Admixture-related differences in linkage disequilibrium blocks surrounding *CTLA4* may also alter the functional impact of rs231775 across populations.

While most research has focused on the transmembrane CTLA-4 isoform as a regulator of T-cell activation, the sCTLA-4 isoform also plays an important immunoregulatory role; elevated sCTLA-4 levels have been described in several autoimmune diseases [29], such as systemic lupus erythematosus [30], myasthenia gravis [31], diffuse cutaneous systemic sclerosis [32], autoimmune thyroid diseases [33], celiac disease [34], and psoriasis [35]. Increased sCTLA-4 concentrations may compete with the CTLA-4 transmembrane isoform for binding to B7 ligands, potentially reducing inhibitory signaling during T cell activation and perpetuating inflammation [36,37]. Thus, sCTLA-4 may function as a double-edged regulator, either dampening immune activation through ligand sequestration or, paradoxically, interfering with effective inhibitory checkpoint signaling depending on the inflammatory context.

In our study, however, sCTLA-4 levels were significantly lower in patients than in controls. This contrasts with prior evidence reporting elevated levels in psoriasis [38]. When stratified by genotype, individuals carrying the GG genotype exhibited higher sCTLA-4 concentrations in both study groups. However, the differences were not statistically significant, consistent with a previous study [38]. Reduced sCTLA-4 levels may reflect diminished immunoregulatory capacity, potentially contributing to enhanced activation, proliferation, and survival of autoreactive T cells. The decreased sCTLA-4 levels observed in our patients may also indicate reduced transcription or alternative splicing of the soluble isoform in activated T cells, which is consistent with evidence of impaired Treg function in psoriasis. Such a deficiency could contribute to unchecked activation of Th1/Th17 pathways, reinforcing the proinflammatory milieu characteristic of the disease [39]. Alternatively, chronic inflammatory signaling may alter post-translational processing or proteolytic shedding of CTLA-4, thereby reducing circulating sCTLA-4 despite ongoing immune activation.

Importantly, no significant differences in age or sex distribution were observed between groups, minimizing the likelihood that demographic variables influenced the immunological findings. Age is known to modulate immune homeostasis through mechanisms such as thymic involution, reduced naïve T-cell output, and increased activation of memory T-cell subsets, thereby altering the regulatory balance between co-stimulatory and co-inhibitory pathways [40]. However, current evidence does not consistently demonstrate a direct age-related increase in CTLA-4 expression or circulating sCTLA-4 levels; rather, age appears to influence the broader immunological context in which CTLA-4 operates, particularly under chronic inflammatory conditions or inflammaging [41]. In our cohort, the absence of age imbalance between groups supports the interpretation that the reduced sCTLA-4 levels observed in patients reflect disease-specific dysregulation of the CTLA-4 pathway rather than demographic confounding. Furthermore, correlation analysis revealed that although disease duration was positively associated with PASI score, sCTLA-4 levels were not correlated with PASI or with any other clinical or anthropometric variables, including age, BMI, or disease duration. This lack of association suggests that circulating sCTLA-4 does not reflect current disease severity or cumulative inflammatory burden in this cohort. Instead, reduced sCTLA-4 levels in patients may represent an underlying immunoregulatory alteration rather than a marker of clinical activity. These findings support the notion that CTLA-4 pathway dysregulation in PP may operate independently of conventional clinical parameters and may reflect a basal checkpoint imbalance rather than dynamic inflammatory fluctuations. Nevertheless, future studies incorporating multivariable modeling could further clarify the independent contribution of CTLA-4-mediated immune regulation to psoriasis pathogenesis.

The discrepancy between our findings and previous reports showing increased sCTLA-4 in psoriasis may reflect differences in population structure, local ancestry, treatment exposure, or disease chronicity. Persistent inflammation can modulate proteolytic shedding and alternative splicing of *CTLA4* transcripts, potentially decreasing circulating sCTLA-4 despite active disease [42]. Moreover, rs231775 alone does not fully capture the regulatory variability within the *CTLA4* locus; promoter variants, the CT60 haplotype, and splicing polymorphisms exert stronger influence on sCTLA-4 production [43]. Therefore, the absence of association observed for rs231775 does not exclude a role for the CTLA-4 pathway in psoriasis pathogenesis. Comprehensive haplotypic analyses and functional assays assessing *CTLA4* expression in T-cell subsets would provide a more complete understanding of checkpoint dysregulation in this disease.

This study has limitations that must be acknowledged. First, although the sample size was estimated a priori, the study may still be underpowered to detect modest effect sizes, which are typical of single-variant associations in complex traits. Second, the cross-sectional design precludes determining whether altered sCTLA-4 levels precede or result from disease activity. Finally, additional *CTLA4* variants and haplotypes were not evaluated, limiting the ability to interpret the genetic contribution to sCTLA-4 production fully. Longitudinal studies that incorporate treatment-naïve patients and functional immune profiling would be valuable for clarifying the temporal and mechanistic relationship between CTLA-4 dysregulation and psoriasis severity.

## 4. Materials and Methods

### 4.1. Study Population

This case–control study included 204 patients with a clinical and histopathological diagnosis of PP and 214 controls. The patients were diagnosed by a dermatologist, enrolled in the Dermatological Institute of Jalisco “Dr. José Barba Rubio”, part of the Mexican Ministry of Health in Guadalajara, Jalisco, Mexico. The control group consisted of clinically healthy individuals living in the same geographic regions as patients and of similar sex and age to PP, and without a family history of psoriasis. Sample collection was conducted between January 2022 and January 2025.

Sample size was estimated a priori for an unmatched case–control study using previously reported data for this SNP in a Mexican population [44]. The calculation was based on the minor allele frequency, assuming a two-sided 95% confidence level, 80% power, and a 1:1 case–control ratio, using the Fleiss method with continuity correction. Both study groups consisted of individuals with a genealogical history of at least three generations residing in Western Mexico, all over 18 years of age, without a diagnosis of chronic degenerative, infectious, autoimmune, or neoplastic disease at the time of enrollment. Blood samples were drawn from all participants by venipuncture into EDTA tubes and into anticoagulant-free tubes after at least 12 h of fasting.

This study was approved by the local Research and Ethics Committee at the Dermatological Institute of Jalisco “Dr. José Barba Rubio” (State Research Registry 82/IDJ-JAL/2022) and conducted in accordance with the ethical statement of the Declaration of Helsinki [11]. Written informed consent was obtained from all subjects participating in the study.

### 4.2. Genotyping of the +49 A>G Variant

**Figure 3:**
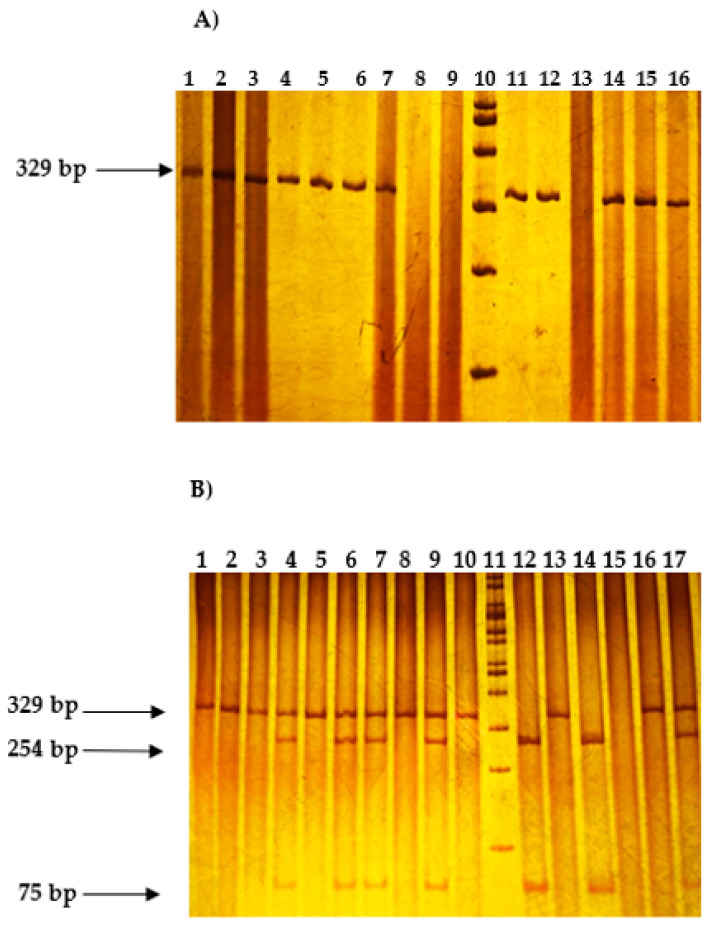
Genotype identification of the +49 A>G *CTLA4* variant. Representative polyacrylamide gel electrophoresis images are shown. (**A**) PCR products. Lane 10: 100-bp molecular weight marker. The 329-bp band corresponds to the amplified polymorphic fragment. (**B**) Restriction fragment length polymorphism (RFLP) products obtained after digestion with *ApeKI*. Genotypes were identified as follows: lanes 1, 2, 3, 5, 8, 10, 13, and 16 (AA genotype); lanes 4, 6, 7, 9, and 17 (AG genotype); lanes 12 and 14 (GG genotype). Lane 11: 100-bp molecular weight marker. Samples with unclear band patterns were re-analyzed in duplicate using an independent electrophoresis run.

Genomic DNA was extracted from peripheral blood using Miller’s modified salting-out technique [12]. The polymorphic fragment was amplified by endpoint polymerase chain reaction (PCR). The +49 A>G variant of the CLTA4 gene was analyzed by polymerase chain reaction-restriction fragment length polymorphism (PCR-RFLP). The following primers were used to obtain a DNA fragment of 329 bp: forward of 5′-CCACGGCTTCCTTTCTCGTA-3′ and reverse 5′-AGTCTCACTCACCTTTGCAG-3′ (Figure 3A). The final reaction volume was 15 μL, containing 0.3 mM primers (Oligo T4, Irapuato, Gto Mexico), 0.15 U/μL Taq DNA polymerase, 1X Buffer A, 1.5 mM MgCl_2_, and 1 mM deoxynucleotide triphosphates dNTPs (Vivantis Technologies. Sdn. Bhd., Oceanside, CA, USA), and 100 ng of DNA as a substrate. The amplification protocol was initial denaturation at 95 °C for 2 min, followed by 30 cycles consisting of denaturation at 94 °C for 30 s, annealing at 65 °C for 30 s, and elongation at 72 °C for 30 s, with a final elongation at 72 °C for 2 min.

Genotyping of PCR products was performed by restriction fragment length polymorphism (RFLP) analysis using the thermostable restriction enzyme *ApeKI* in CutSmart buffer (New England BioLabs, Inc., Ipswich, MA, USA) for 16 h at 75 °C, according to the manufacturer’s recommendations.

Three genotypes were obtained: wild-type homozygous AA (329 bp fragment), heterozygous AG (329, 254, and 75 bp fragments), and polymorphic homozygous GG (254 and 75 bp fragments). The PCR products and restriction fragments were visualized by vertical electrophoresis on 6% polyacrylamide gels and stained with 0.2% silver nitrate (Figure 3B). To ensure genotyping accuracy, a random subset of samples (10%) was independently re-genotyped, yielding 100% concordance between assays.

### 4.3. Quantification of sCTLA-4 Serum Levels

sCTLA-4 serum levels were quantified in a randomly selected subset of participants (n = 40 per group) using the commercial Human sCD152/CTLA-4 ELISA Kit (Invitrogen™ Life Technologies, Carlsbad, CA, USA), according to the manufacturer’s instructions. All samples were analyzed in duplicate to ensure measurement reliability.

The assay sensitivity was 0.13 ng/mL, with a dynamic detection range of 0.16–10.0 ng/mL. Values at or below the assay detection limit were recorded as the lower limit of detection for statistical analysis.

### 4.4. Statistical Analysis

Statistical analysis was performed using Stata v16.0 and GraphPad Prism v8.0. For descriptive analysis, categorical variables were reported as frequencies, continuous variables with nonparametric distributions were reported as medians and 5th–95th percentile ranges, and parametric variables were reported as means ± standard deviations (SDs). Genotypic and allelic frequencies were obtained by direct counting. Hardy–Weinberg equilibrium was assessed in the control group by the Chi-square (χ2) test. The association analysis between genotypes and PP was estimated using the odds ratio (OR) with 95% confidence interval (CI). The Mann–Whitney U test was used for nonparametric quantitative comparisons, and the Kruskal–Wallis test, followed by Dunn’s multiple comparison test, was used for multi-group analyses. Statistical significance was set at *p* < 0.05.

## 5. Conclusions

In conclusion, our results show that the +49 A>G variant of the *CTLA4* gene (rs231775) is not associated with susceptibility to PP in the Mexican mestizo population. Despite the lack of a genetic association, patients had significantly lower serum sCTLA-4 levels than controls. This reduction may reflect impaired immunoregulatory function, potentially contributing to the heightened T-cell activation and inflammatory responses characteristic of psoriasis.

These results suggest that alterations in sCTLA-4 expression, rather than the +49 A>G variant itself, may play a more relevant role in the immunopathogenesis of psoriasis within this population. Future studies with larger sample sizes, evaluation of additional *CTLA4* variants, haplotype analyses, and assessments of gene–environment and gene–gene interactions are warranted to clarify the contribution of the *CTLA4* pathway to psoriasis. Moreover, integrating functional assays and longitudinal analyses could help determine whether sCTLA-4 may serve as a biomarker of immune dysregulation or disease activity.

## Figures and Tables

**Table 1:** Clinical and demographic characteristics of the study groups.

Characteristics	CS (n = 214)	PP (n = 204)	*p*-Value
Age (years) (mean ± SD)	44.7 ± 12.06	46.8 ± 14.48	0.078
Gender, n (%)			
Female	105 (49)	96 (47)	0.77
Male	109 (51)	108 (53)	
Disease duration (years) (mean ± SD) Age of onset (years) (mean ± SD)	-	9 ± 10 38 ± 18	
Topical treatment (exfoliants) n (%)		30 (15)	

Abbreviations: PP, plaque psoriasis; CS, control subjects; SD, Standard Deviation; n, sample size.

**Table 2:** Genotype and allele frequencies of +49 A>G *CTLA4* variant in cases and controls.

Variant rs231775	CS (n = 214) n (%)	PP (n = 204) n (%)	OR (CI 90%)	*p*-Value
Genotypes				
AA ^a^	66 (31)	72 (35)	1	-
AG	114 (53)	99 (49)	0.80 (0.51–1.25)	0.30
GG	34 (16)	33 (16)	0.89 (0.47–1.66)	0.69
Alleles				
A ^a^	246 (57)	243 (60)	1	-
G	182 (43)	165 (40)	0.92 (0.69–1.22)	0.54
Genetic models				
Dominant				
AA ^a^	66 (31)	72 (35)	1	-
AG+GG	148 (69)	132 (65)	0.82 (0.53–1.25)	0.33
Recessive				
AA+AG ^a^	180 (84)	171 (84)	1	-
GG	34 (16)	33 (16)	1.02 (0.58–1.78)	0.93

^a^ Genotype or allele of reference. *p*-values were calculated using the Chi-square test. Abbreviations: PP, plaque psoriasis; CS, control subjects; OR, odds ratio; CI, confidence interval.

**Table 3:** Spearman correlation analysis among clinical variables in PP.

Variable	Age ρ (*p*-Value)	BMI ρ (*p*-Value)	PASI ρ (*p*-Value)	sCTLA-4 ρ (*p*-Value)	Disease Duration ρ (*p*-Value)
Age	—	0.18 (0.49)	0.42 (0.093)	0.18 (0.49)	0.21 (0.42)
BMI	0.18 (0.49)	—	0.36 (0.16)	−0.12 (0.65)	0.09 (0.73)
PASI	0.42 (0.093)	0.36 (0.16)	—	0.09 (0.73)	0.51 (0.036)
sCTLA-4	0.18 (0.49)	−0.12 (0.65)	0.09 (0.73)	—	0.21 (0.42)
Disease Duration	0.21 (0.42)	0.09 (0.73)	**0.51 (0.036)**	0.21 (0.42)	—

Values are presented as Spearman’s correlation coefficient (ρ) with corresponding two-tailed *p*-values in parentheses. Statistically significant correlations (*p* < 0.05) are highlighted in bold in the manuscript text. BMI: Body Mass Index; PASI: Psoriasis Area and Severity Index; sCTLA-4: soluble cytotoxic T-lymphocyte-associated antigen 4.

## Data Availability

All relevant data supporting the findings of this study are included within the article. Additional data may be available from the corresponding author upon reasonable request.

## Abbreviations

The following abbreviations are used in this manuscript:

PP	Plaque psoriasis
CS	Control subjects
CTLA-4	Cytotoxic T-lymphocyte-associated antigen 4
sCTLA-4	Soluble cytotoxic T-lymphocyte-associated antigen 4
Treg	Regulatory T cells
Th17	T helper 17 cells
Th1	T helper 1 cells
SNV	Single-nucleotide variant
PCR	Polymerase chain reaction
PCR-RFLP	Polymerase chain reaction–restriction fragment length polymorphism
OR	Odds ratio
CI	Confidence interval
PASI	Psoriasis Area and Severity Index
BMI	Body mass index
